# Supplementary material for: Optimization of modularity during development to simplify walking control across multiple steps
Source: Front Neural Circuits. 2024 Jan 26;17:1340298. doi: 10.3389/fncir.2023.1340298 (PMC10853381; doi:10.3389/fncir.2023.1340298)
Supplement: Supplementary file 1 [file Table_1.docx]

**Supplementary material**

Table S1. Individual values regarding EMG variability and features of modularity. Each index was computed in Primary Condition (PC) and in Control Condition (CC). CC consists of computational control for adults and of experimental control for toddlers (Figure 3).

|  | IEV | | VAF | | Z-t. VAF | | Module nb | | IRV | | IRS | |
| --- | --- | --- | --- | --- | --- | --- | --- | --- | --- | --- | --- | --- |
|  | PC | CC | PC | CC | PC | CC | PC | CC | PC | CC | PC | CC |
| Adult 1 | 0.06 | 0.06 | 0.65 | 0.65 | 1.12 | 1.12 | 6 | 6 | 3.27 | 0.62 | 0.62 | 0.62 |
| Adult 2 | 0.06 | 0.06 | 0.74 | 0.74 | 1.30 | 1.30 | 5 | 5 | 4.35 | 0.61 | 0.59 | 0.58 |
| Adult 3 | 0.06 | 0.07 | 0.70 | 0.69 | 1.20 | 1.19 | 5 | 5 | 4.74 | 0.64 | 0.63 | 0.63 |
| Adult 4 | 0.05 | 0.06 | 0.81 | 0.80 | 1.48 | 1.45 | 4 | 4 | 5.42 | 0.55 | 0.56 | 0.55 |
| Adult 5 | 0.05 | 0.05 | 0.80 | 0.80 | 1.45 | 1.43 | 4 | 4 | 5.26 | 0.59 | 0.60 | 0.59 |
| Adult 6 | 0.06 | 0.06 | 0.71 | 0.70 | 1.23 | 1.22 | 5 | 5 | 3.33 | 0.62 | 0.65 | 0.64 |
| Adult 7 | 0.06 | 0.07 | 0.73 | 0.72 | 1.27 | 1.25 | 5 | 5 | 5.04 | 0.59 | 0.60 | 0.59 |
| Adult 8 | 0.06 | 0.06 | 0.76 | 0.75 | 1.34 | 1.33 | 4 | 4 | 4.92 | 0.58 | 0.57 | 0.58 |
| Adult 9 | 0.04 | 0.05 | 0.77 | 0.76 | 1.38 | 1.33 | 4 | 4 | 3.65 | 0.60 | 0.62 | 0.60 |
| Adult 10 | 0.05 | 0.05 | 0.74 | 0.74 | 1.29 | 1.29 | 5 | 5 | 3.10 | 0.64 | 0.64 | 0.65 |
| Adult 11 | 0.04 | 0.05 | 0.71 | 0.69 | 1.22 | 1.20 | 5 | 5 | 2.73 | 0.63 | 0.64 | 0.62 |
| Adult 12 | 0.05 | 0.06 | 0.77 | 0.75 | 1.36 | 1.33 | 4 | 4 | 5.13 | 0.60 | 0.61 | 0.60 |
| Toddler 1 | 0.11 | / | 0.56 | / | 0.97 | / | 7 | / | 7.25 | / | 0.43 | / |
| Toddler 2 | 0.12 | / | 0.70 | / | 1.20 | / | 6 | / | 9.69 | / | 0.48 | / |
| Toddler 3 | 0.13 | 0.11 | 0.51 | 0.62 | 0.89 | 1.07 | 7 | 7 | 7.00 | 6.20 | 0.38 | 0.45 |
| Toddler 4 | 0.12 | 0.11 | 0.62 | 0.58 | 1.06 | 0.99 | 6 | 8 | 7.53 | 4.97 | 0.44 | 0.39 |
| Toddler 5 | 0.14 | 0.11 | 0.63 | 0.71 | 1.08 | 1.24 | 6 | 5 | 9.13 | 13.57 | 0.39 | 0.38 |
| Toddler 6 | 0.12 | 0.10 | 0.50 | 0.56 | 0.87 | 0.97 | 8 | 8 | 5.46 | 4.28 | 0.35 | 0.43 |
| Toddler 7 | 0.12 | 0.11 | 0.53 | 0.58 | 0.92 | 1.01 | 7 | 7 | 5.65 | 5.92 | 0.35 | 0.44 |
| Toddler 8 | 0.14 | 0.12 | 0.52 | 0.54 | 0.91 | 0.94 | 8 | 7 | 5.64 | 6.66 | 0.36 | 0.37 |
| Toddler 9 | 0.13 | 0.11 | 0.52 | 0.49 | 0.91 | 0.87 | 8 | 9 | 5.26 | 3.32 | 0.38 | 0.35 |
| Toddler 10 | 0.12 | 0.13 | 0.64 | 0.59 | 1.10 | 1.02 | 6 | 7 | 8.84 | 7.80 | 0.40 | 0.35 |
| Toddler 11 | 0.11 | 0.11 | 0.53 | 0.58 | 0.93 | 1.00 | 7 | 7 | 6.37 | 6.03 | 0.38 | 0.42 |
| Toddler 12 | 0.13 | 0.10 | 0.65 | 0.59 | 1.11 | 1.02 | 6 | 6 | 9.36 | 8.21 | 0.43 | 0.40 |
